# Revealing biomechanical vulnerabilities in oral cancer cells using 3D coculture platform and low-frequency ultrasound

**DOI:** 10.1016/j.mtbio.2026.103375

**Published:** 2026-06-19

**Authors:** Rashmita Luha, Gomathi Sankar, Akshay Kumar, Alka Kumari, Ketan Kulkarni, Rudra Pratap, Aravind Kapali, Ajay Tijore

**Affiliations:** a Department of Bioengineering, Indian Institute of Science, Bangalore, 560012, India; b Centre for Nano Science and Engineering, Indian Institute of Science, Bangalore, 560012, India; c Plaksha University, Mohali, 140306, India; d Department of Surgical Oncology, M. S. Ramaiah Medical College & Hospitals, Bangalore, 560054, India

**Keywords:** Low-frequency ultrasound (LFU), Mechanical forces, Oral cancer, Apoptosis, Tropomyosin 2.1, MicroRNA-21

## Abstract

Achieving selective and efficient targeting of cancer cells while preserving the normal cells remains a crucial obstacle in oral cancer therapies, which compromises patients' quality of life. Here, we present a non-invasive approach using low-frequency ultrasound (US) to exploit the biomechanical vulnerabilities of patient-derived oral cancer cells, thereby promoting selective apoptosis. Cancer cells are subjected to optimized US parameters, revealing selective induction of cancer cell apoptosis (mechanoptosis) without harming normal cells. Reduced expression of the mechanosensory protein Tropomyosin 2.1 (Tpm2.1) in oral cancer cells, due to elevated levels of MicroRNA-21 (miR-21), correlates with heightened sensitivity to US treatment. Furthermore, mechanistic studies demonstrate that US disrupts actomyosin contractility by disassembling myosin IIA fibers, thereby impairing the migration and invasion of cancer cells. Remarkably, a study using an elastomeric platform for coculture of patient-derived cancer and cancer-associated fibroblast (CAF) cells shows a significant reduction in CAFs’ ability to infiltrate and compartmentalize the tumor core as well as encapsulate the tumor upon US treatment. Our findings suggest that an ultrasound-based strategy could be used to target the oral tumor microenvironment to augment existing cancer treatment and may open avenues for broader application in superficial malignancies.

## Highlights

• LFU selectively induces mechanical force-mediated apoptosis (mechanoptosis) in patient-derived oral cancer cells.
• The 3D coculture platform reveals that LFU disrupts cancer cells–CAF interactions that normally form stromal barrier around the tumor core.

## Graphical abstract

**Figure:**
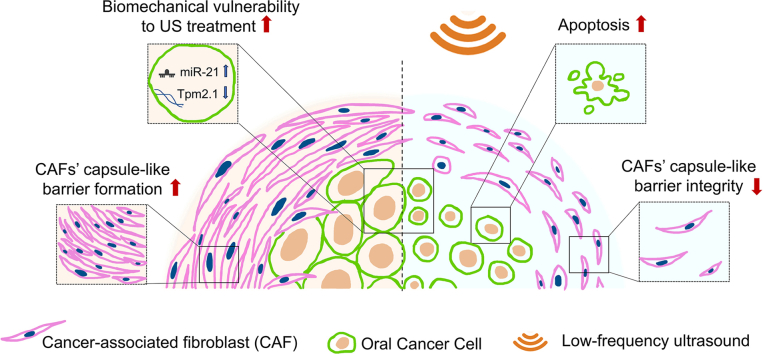

## 1 Introduction

Oral cancer is the most prevalent cancer type of the head and neck squamous cell carcinoma (HNSCC), developing in the oral cavity epithelium [1]. The incidence and mortality rates continue to rise in developing countries like India, which account for more than one-third of the total oral cancer burden globally, owing to the heavy consumption of tobacco, including betel quid, areca nut chewing and alcohol [2,3]. Despite advances in current treatment therapies such as surgery, radiotherapy, and chemotherapy, the survival rates over the last five decades have remained unchanged, suggesting the clinical inefficacy of these therapies [4]. The severe side effects associated with conventional radio- and chemotherapy, for example, are constrained by the nonselective targeting that damages both tumor and adjacent normal tissues alike, which ultimately undermines their efficacy. In addition, due to heterogeneity, patients show significant variations in response to radio- and chemo-therapy, leading to unnecessary exposure to radiation and chemotherapeutic drugs, developing therapy resistance, and cancer relapse [5,6]. Consequently, there is an urgent need to develop selective therapies that can overcome these challenges by harnessing the distinct biomechanical properties of cancer and normal cells, thus offering great potential to minimize side effects and to enhance therapeutic efficacy [7].

Ultrasound strategies such as high-intensity focused ultrasound (HIFU) [8,9] and high-intensity pulsed ultrasound (HIPU) [10] are being used for cancer therapies, including prostate [11], pancreatic [12] and breast cancer [13]. The main principle of these modalities is mechanical and thermal ablation of tumor cells via coagulation necrosis [13,14]. However, the clinical efficacy is limited by the dissipation of thermal energy into surrounding normal tissues, leading to normal cell death and a subsequent inflammatory response [15,16]. Intriguingly, emerging reports have shown that low-frequency ultrasound (US) promotes selective calcium-mediated apoptosis without harming normal cell function or growth [[16], [17], [18], [19], [20], [21]]. In particular, molecular mechanistic studies demonstrated that US treatment activated mechanosensitive Piezo1 channels, causing calcium influx into cancer cells, followed by activation of the calpain-dependent apoptotic pathway. The underlying mechanism of this selectivity and sensitivity is due to the difference in the biomechanical properties of cancer cells and normal cells [[22], [23], [24]]. Interestingly, this mode of therapeutic US is approved for human use [[25], [26], [27]] and is based on the induction of the apoptotic pathway rather than thermal effects [17,21]. Hence, it suggests the potential to develop a non-invasive, low-frequency US-based cancer treatment.

Given the mechanical vulnerability of cancer cells to physiologically relevant mechanical forces generated by US treatment, this study therefore seeks to investigate the effects of US-generated mechanical forces on the *ex vivo* survival of patient-derived oral cancer cells and on tumor organization integrity using a 3D elastomeric coculture platform. Interestingly, significant oral cancer cell apoptosis was observed upon US treatment at optimized parameters that do not harm normal cells. In addition, myosin-mediated contractility was drastically reduced upon US treatment, impairing cancer cells' ability to invade and contract collagen gel. Furthermore, the time-lapse imaging of the *in vitro* platform with cancer cells and CAFs coculture reveals that the CAF-formed capsule-like barrier, which excludes immune cells and cancer therapies from infiltrating the tumor core, is remarkably disrupted by US treatment. Altogether, the selective killing of cancer cells, along with the disruption of the stromal barrier surrounding the tumor core, compromises the CAF's capsule-like barrier integrity, highlight the potential of developing novel US-mediated therapies to overcome existing common therapeutic limitations.

## 2 Materials and methods

### 2.1 Clinical samples and ethical considerations

Oral tumor samples were obtained from the Department of Surgical Oncology, M. S. Ramaiah. Medical College & Hospitals (Bangalore, India) under informed consent and approval of the. M. S. Ramaiah Hospital Ethics Committee (Reg. No. ECR/215/Inst/KA/2013/RR-22) and. Institutional Human Ethics Committee of the Indian Institute of Science (IISc, Bangalore, India). All experiments were performed on tumor samples collected from January 2023 to December 2025, in accordance with institutional biosafety guidelines and with patient consent.

### 2.2 Cell isolation

The cells were isolated from fresh tumor samples and characterized at the Mechanobiologics lab, Indian Institute of Science. The present protocol and reagents for isolation of normal and oral cancer cells from patient samples have been modified from previously described protocols [28,29]. Briefly, the freshly resected patient tumor samples and normal biopsy samples were transported in transport medium composed of Minimum Essential Medium Eagle (MEM, Himedia), 10% Fetal Bovine Serum (FBS, Gibco, Origin: Brazil), 2% Penicillin-Streptomycin (5000 U/mL Pen-Strep, Gibco) and 2% Amphotericin (250 μg/mL, Himedia) in the ice box and processed within 1-2 h post-surgery. The samples were thoroughly washed with PBS (1X, Gibco), containing 2% Penicillin-Streptomycin and Amphotericin, three times with gentle rocking for 3 min each. The sample was then transferred to 90 mm petri dishes (Genaxy) for mincing along with a little volume of digestion media (1% FBS, 2% Penicillin-Streptomycin and Amphotericin, 0.4 mg/mL Collagenase Type 1 (Gibco)). Debris, blood vessels, fats and necrotic tissues were removed, and the sample was then minced into a fine slurry using surgical blades (B.P. Blade No. 11). The slurry was transferred to the digestion media and allowed to digest in the hybridization chamber maintained at 37 0C and 80 rpm for 1-2 h, depending on the tumor samples. After complete digestion, a significant decrease in the pellet was observed. The digested sample was centrifuged at 1.3 × 10^3^ rpm for 3 min, and the pellet was resuspended in growth medium (10% FBS, 2% Penicillin-Streptomycin and Amphotericin, 20 ng/mL EGF, 1 ng/mL Insulin and 100 ng/mL Hydrocortisone). The supernatant was centrifuged again at 1.8 × 10^3^ rpm for 5 min to obtain cancer-associated fibroblasts, which were then resuspended in growth medium. The resuspended solution was transferred to a collagen-coated 35 mm culture dish, incubated at 37 ^0^C with 5% CO_2_, and grown for two weeks in a growth medium with media changes every alternate day. Differential trypsinization was performed to remove CAFs until the epithelial cells became a predominant population in the petri dishes. Fig. S1 shows the schematic of the steps involved in isolating primary oral cancer cells from patient tumor samples. A combination of TrypLE Express Enzyme (1X, Gibco) and Trypsin from porcine pancreas (Sigma) at a 1:1 ratio was used for cell detachment. Table S1 shows the patients’ information and the experimental details performed.

### 2.3 Cell culture

The MCF 10A cells (non-transformed human mammary epithelial cell line) were a gift from Dr. Medhavi Vishwakarma's lab (IISc Bangalore). The cells were maintained in Dulbecco's modified Eagle's medium (DMEM–F12, Gibco) supplemented with Human EGF Recombinant Protein (20 ng/mL, Gibco), Hydrocortisone (0.5 mg/mL, Sigma-Aldrich), Cholera toxin (100 ng/mL; Sigma-Aldrich), Insulin (10 μg/mL, Sigma-Aldrich), with 10% Fetal Bovine Serum (FBS, Gibco, Origin: Brazil) and Pen-Strep (100 μg/mL, Gibco) at 37 °C in a humidified incubator with 5% CO_2_. The MDA-MB-231 cells were obtained from Dr. Ramray Bhat (IISc Bangalore) and were cultured in high-glucose Dulbecco's Modified Eagle Medium (DMEM, Gibco) supplemented with 10% fetal bovine serum (FBS, Sigma-Aldrich) and Pen-Strep (100 μg/mL, Gibco) at 37 °C in a humidified incubator with 5% CO_2_. 1:1 TrypLE Express Recombinant Enzyme (Gibco) and Trypsin-EDTA (Gibco) were used for cell subculturing and experiments. Early-passage primary oral cancer cells (passage 1–2) cultured on 50 μg/mL collagen-I, rat tail (Gibco) coated dishes were used in all experiments.

### 2.4 Ultrasound treatment

The custom-made US device was used to apply US pressure on cell samples (Fig. S2A). The device fabrication and functioning were reported previously in the papers [[19], [20], [21]]. Briefly, the cells were treated with ultrasound generated by the Langevin piezoelectric transducer (APC 90–4050). The transducer was bonded to a steel container and placed on a custom-made 3D printed platform with adjustable XYZ planes to facilitate US treatment of cell cultures. The culture dishes were placed on a 3D-printed epoxy tray, which supported the cell dishes and kept them half submerged in the degassed water in the steel container. The transducer was actuated at a 50% duty cycle and 39 kHz frequency for 2 h, unless otherwise specified, using Ultrasound Driver (PDU 210). The pressure experienced on the glass-bottom dishes (Cellvis 35 mm dish with 10/20 mm glass bottom) was measured with a hydrophone connected to a USB oscilloscope (Digilent 410-321) by submerging it in culture medium, followed by replacing it with the experimental samples sealed with parafilm to avoid possible contamination and water entry during US treatment. The voltage applied to the transducer was adjusted to achieve the desired pressure levels. Fig. S2B shows the temperature and pressure values over 2 h of US treatment. Following treatment, samples were returned to the incubator for further experiments or fixed for immunostaining. In all experiments, the US treatment duration is 2 h, unless otherwise mentioned.

### 2.5 Immunocytochemistry

Cells were washed with phosphate-buffered saline (PBS, Gibco) and fixed in 4% paraformaldehyde (PFA, ThermoFisher Scientific) for 10 min at room temperature (RT) and permeabilized with 0.5% Triton X-100 (Sigma-Aldrich) for 5 min at RT. Cells were washed thrice with PBS and blocked in 4% bovine serum albumin (BSA, Signa-Aldrich) for 1 h at RT before being incubated for 1 h at 37 °C with primary antibodies: rat anti-cytokeratin 8 (CK8, 1:300, Sigma-Aldrich, MABT329), mouse anti-α-smooth muscle actin (α-SMA, 1:400, Sigma-Aldrich, A2547), mouse anti-paxillin (1:300, BD Transduction Laboratories, 610052), rabbit polyclonal anti-myosin IIA non-muscle (1:400, Sigma, M8064), mouse anti-tropomyosin, Tm2 (1:300, DSHB, CG1). Cells were then incubated at RT with secondary antibodies for 2 h: goat anti-rat Alexa Fluor 680 (1:400, Invitrogen, A21096), goat anti-mouse Alexa Fluor 488 (1:400, Invitrogen, A11029), goat anti-mouse Alexa Fluor 568 (1:400, Invitrogen, A11004), goat anti-rabbit Alexa Fluor 488 (1:400, Invitrogen, A11034), goat anti-rabbit Alexa Fluor 546 (1:400, Invitrogen, A12381). For F-actin staining, cells were incubated with Phalloidin 594 (1:400, Invitrogen) for 1 h. Hoechst dye (1:1000, Invitrogen) was used to stain the cell nucleus. The samples were then washed with PBS, and immunofluorescence images were captured using a fluorescence microscope with a 60× oil immersion objective (EVOS M5000 imaging system, Invitrogen).

### 2.6 Transfection

Cells were transiently transfected with mApple-Paxillin plasmid and siRNA using Lipofectamine™ 3000 transfection reagent (Invitrogen) in Opti-MEM (serum-free, Gibco) as per manufacturer's instructions. mApple-Paxillin plasmid was transfected to enable fluorescent visualization of focal adhesions, and Tpm2.1 siRNA was transfected to achieve targeted knockdown of Tpm2.1 expression for downstream functional assays. Experiments were performed after 48 h of incubation for higher transfection efficiency.

### 2.7 Collagen gel contraction assay

Collagen I solution (Collagen I rat tail, Gibco) was prepared at 2.5 mg/mL in 0.1% acetic acid. Cells were mixed with the collagen solution and the final solution was constituted to 1 mg/mL 1 N NaOH was used to adjust the pH of the 1 mg/mL collagen solution, which was then plated in a 96-well plate in triplicate. The cells-embedded collagen solution was incubated for 1 h at 37 ^0^C to polymerize, followed by releasing the collagen gel from the sides of the 96-well plate with a 2.5 μl pipette tip. Fresh complete media was added, and the collagen gel was further incubated for 48 h. Collagen gel size change was monitored by imaging every 24 h. The areas of the collagen gel over time were analyzed using ImageJ.

### 2.8 3D migration assay

Oral cancer cells were seeded in 96-well plates (3000 cells/well) pre-coated with poly- (2-hydroxyethyl methacrylate) (PolyHEMA, Sigma) to create low-adhesion for spheroid formation. The cells were cultured for 24 h in defined medium supplemented with Matrigel (Corning) at 37 ^0^C. These spheroids were then embedded in a collagen type I (Gibco) and polymerized at 37 ^0^C. Cell migration from embedded spheroids was observed with and without US treatment using live imaging on confocal microscopy.

### 2.9 3D invasion assay

Collagen I solution (Collagen I rat tail, Gibco) was prepared at 3 mg/mL in 0.1% acetic acid. Patient-derived oral cancer cells seeded in a 96-well plate at a density of 8 × 10^3^ cells per well after treatment were incubated with Hoechst (1:1000) for 10 min. Working solution of collagen (2.5 mg/mL) in serum-free DMEM was prepared and added to the 96-well plate (100 μL per well). Collagen was incubated for 30 min at 37 ^0^C to polymerize, and MEM with 10% FBS was added on top. Cells were allowed to invade for 24 h. Z-stack images was taken at 0 μm and 60 μm height of collagen matrix to check the invasion in untreated and US-treated samples.

### 2.10 Apoptosis assay

The Annexin V-Alexa Fluor 488 (Invitrogen, A13201) staining solution was used to demarcate dead cells from viable cells, according to the manufacturer's protocol. Annexin V stains only the cells with compromised plasma membrane integrity. Briefly, the cells were incubated with Annexin V at RT for 15 min after 8 h of ultrasound treatment. Quantification of the apoptotic cells was performed by measuring the number of cells stained with Annexin V.

### 2.11 Immunoblotting

Total cellular protein from oral cancer or MCF 10A cells was extracted using a cocktail of RIPA buffer (ThermoFisher Scientific) and protease inhibitor cocktail (Roche). The cell suspension mixture was then centrifuged at 12000× g for 20 min, and maintained at 4 °C, and the supernatant was collected. A solution mixture of 30 μg protein extract, β-mercaptoethanol and lammeli buffer (Bio-Rad, 1610737) was allowed for denaturation at 95 °C for 10 min followed by loading to 10% acrylamide gel (TGX Stain-Free FastCast, Bio-Rad, 1610182) prepared as per the manufacturer's instructions. A Trans-Blot Turbo Transfer System was used to transfer the gel to a nitrocellulose membrane. After blocking with 5% BSA in Tris-buffered saline with Tween-20 (TBST) for 2 h, the membrane was incubated with primary antibodies overnight at 4 °C: mouse α-tubulin (1:2000, Invitrogen, 62204) or anti-tropomyosin, Tm2 (1:1000, DSHB, CG1). After washing thoroughly, the membranes were then incubated with goat anti-mouse secondary antibody (1:5000, Invitrogen, 31430) for 2 h at RT. The membrane bands were captured with ChemiDoc XRS+ (Bio-Rad) using the built-in Image Lab software.

### 2.12 qRT-PCR

RNA was extracted using the RNeasy Mini Kit (Qiagen, 74104) following the manufacturer's instructions. Reverse transcription was performed using iScript cDNA Synthesis Kit (Bio-Rad, 1708891) as per the manufacturer's instructions. Quantitative real-time PCR was performed using iTaq Universal SYBR Green Supermix (Bio-Rad) according to the manufacturer's instructions in the CFX Duet RT-PCR System (Bio-Rad). Total RNA isolated from MCF 10A was used as a negative control. To evaluate the expression of miR-21, patient-derived oral cancer cells and MCF 10A cells were cultured for 48 h, after which total RNA was isolated. cDNA synthesis was performed with 1 μg of total RNA, followed by qRT-PCR with miR-21 and U6 snRNA primers (Primer sequences mentioned in Table S2. U6 snRNA was used as an internal control, and relative levels of miR-21 were determined using the comparative CT (ΔΔCT) method and represented as relative fold change.

### 2.13 Immunohistochemistry (IHC) analysis

IHC staining was performed to check the spatial distribution of oral cells and CAFs in the patient-derived tumor samples. Briefly, IHC was performed using 4 μm sections of parafilm-embedded oral tumor. Epitope retrieval was performed in Tris-EDTA at 65 °C overnight incubation, followed by 1 N HCl and Trypsin-CaCl_2_ treatment. The slides were blocked with bovine serum albumin (5%) and incubated with primary antibodies: rabbit anti-pan-cytokeratin (anti-PCK, 1:100, Abcam, ab9377) and mouse anti-α-smooth muscle actin (α-SMA, 1:200, Sigma-Aldrich, A2547) for overnight at 4 °C. Sections were washed thoroughly to remove non-specific staining followed by incubation with corresponding horseradish peroxidase (HRP) conjugated secondary antibody for 45 min at RT. Sections were washed and incubated for 1 h in 3,3′-diaminobenzidine (DAB). The sections were counterstained with hematoxylin after washing. Mounted samples were acquired by an Olympus BX53F brightfield microscope.

### 2.14 PDMS well platform and coculture

The PDMS (polydimethylsiloxane) well platform was fabricated using conventional soft lithography. The platform height (⁓200 μm) was achieved via spin coating. A hollow well was created using a 2.5 mm biopsy puncture needle and sealed onto a glass-bottom dish via plasma treatment (Fig. 5B). 300-500 μm-sized tumor aggregates were prepared by digesting tumor samples. These aggregates were labelled with a cell tracker (green) (ThermoFisher Scientific, CellTracker ™ Fluorescent Probes, C2102) as per the manufacturer's instructions and seeded into collagen-coated PDMS wells. After an overnight culture, cell tracker (ThermoFisher Scientific, CellTracker ™ Deep Red, C34565) was used to label primary CAFs as per the manufacturer's instructions and seeded into wells and allowed to grow.

### 2.15 Microscopy and image analysis

Immunofluorescence images were acquired using a 60× oil-immersion objective on a ThermoFisher Scientific EVOS M5000 inverted microscope (Invitrogen). For live imaging, samples were placed on a stage-top incubator and maintained at 37 °C and 5% CO_2_, along with the US setup for treatment. A confocal microscope LEICA TCS SP8 with LAS X (Leica Application Suite X) software, and the time-lapse images were acquired one frame/30 min with 10× air objective, were used for live imaging for 8 h. All the immunofluorescence images, immunoblot analysis, and processing were performed using the freely available ImageJ program. The speed and track of the individual infiltrated CAFs within the tumor were measured by the ImageJ plugin MTrackJ.

### 2.16 Statistical analysis

Statistical analysis was performed using GraphPad Prism (version 8.0). An unpaired two-tailed Student's t-test was used for comparing means of two groups, and for more than one variable, ANOVA was used, followed by Tukey's multiple comparisons. All the data were shown as mean ± standard error of the mean (SEM) unless otherwise specified. Quantification involves data from two or three independent biological experiments with at least two technical replicates per group. For live-cell experiments, each graph shows data from multiple regions of a frame spanning 8 h. Data were considered statistically significant at ∗*p* < 0.05, ∗∗*p* < 0.01, ∗∗∗*p* < 0.001 and ∗∗∗∗*p* < 0.0001.

## 3 Results

### 3.1 Patient-derived oral cancer cells exhibit distinct morphological and phenotypic characteristics

**Fig. 1:**
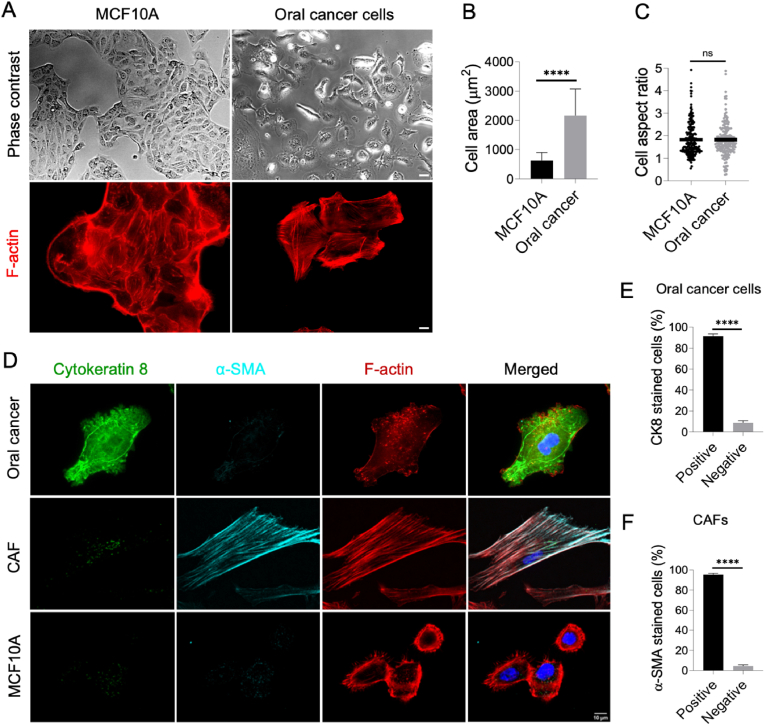
Morphological features of patient-derived oral cancer cells and their characterization. (A) Representative phase-contrast and immunofluorescent images of normal breast epithelial cells (MCF 10A) and oral cancer cells. Scale bar: 50 μm for phase-contrast and 10 μm for immunofluorescent images. (B, C) Bar graph showing the cell area and aspect ratio of MCF 10A and oral cancer cells, respectively. Two-sided, unpaired Student's t-test, n > 100 cells from two different patient samples. (D) Representative immunofluorescence images of primary oral cancer, cancer-associated fibroblast (CAF) and MCF 10A stained with different markers, such as cytokeratin 8 (cancer cell), α-SMA (CAFs) and F-actin (stress fiber). Scale bar: 10 μm. (E, F) Bar graph depicting the number of oral cancer cells and CAFs expressing cytokeratin 8 and α-SMA, respectively. Two-sided, unpaired Student's t-test, n > 100 cells from two different patient samples. In all experiments, ns: non-significant, ∗*p* ​< ​0.05, *∗∗p* ​< ​0.01, *∗∗∗p* ​< ​0.001, and *∗∗∗∗p* ​< ​0.0001.

To investigate the effect of ultrasound treatment on the survival of oral cancer cells, patient-derived tumor samples were collected. Oral cancer cells were isolated using the enzymatic digestion method as described in the ‘experimental method’ section (Fig. S1). To consider the inter- and intra-tumor heterogeneity across patients, cells were isolated from patients with cancer stages I-IV, primarily from the buccal mucosa, gingivobuccal mucosa, and alveolus (Table S1). Oral cancer cells showed a characteristic epithelial-shaped spread morphology similar to the epithelial monolayer growth in normal breast epithelial cells (MCF 10A) (Fig. 1A) and patient-derived normal oral epithelial cells (Fig. S3A). In particular, a 3-fold and 1.8-fold increase in cell area was observed in oral cancer cells compared to normal MCF1 and oral epithelial cells (Fig. 1B and Fig. S3B). However, no changes in the aspect ratio (∼2) were observed in cancer and normal MCF10 and oral epithelial cells (Fig. 1C and Fig. S3C).

Next, isolated cells were characterized using various mature markers of malignant, fibrotic and normal cells (Fig. 1D and Fig. S4). Isolated oral cancer cells (∼90% cells) displayed a distinct expression of cytokeratin 8 (CK8) (Fig. 1E), whereas CAFs and normal MCF 10A cells did not exhibit CK8 expression, thereby differentiating between cancerous and non-cancerous cells [30]. Similarly, isolated CAFs (∼95%) exhibited high α-smooth muscle actin (α-SMA) expression (Fig. 1F), a mature marker of activated fibroblasts, whereas cancer and normal cells lacked α-SMA expression. Overall, our results indicated the successful isolation and expansion of oral cancer cells.

### 3.2 Low-frequency ultrasound reduces cancer cell spreading by disassembling focal adhesions

**Fig. 2:**
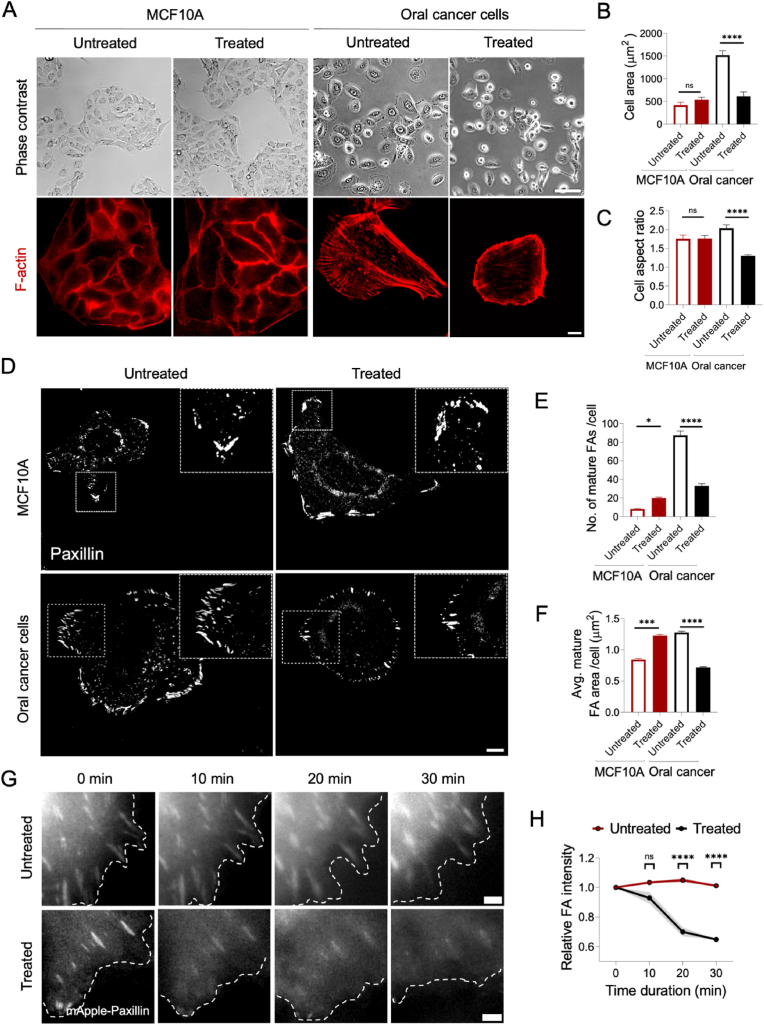
Low-frequency ultrasound inhibits the spreading of oral cancer cells by suppressing focal adhesions. (A) Representative phase-contrast and F-actin images of MCF 10A and oral cancer cells with and without US treatment. Scale bar: 100 μm for phase-contrast and 10 μm for F-actin images. (B, C) Bar graph showing the cell area and aspect ratio with and without US treatment in MCF 10A and oral cancer cells, respectively. Two-way ANOVA followed by Tukey's multiple comparisons test, n > 100 cells from two different patient samples. (D) Representative immunofluorescence images of focal adhesions of MCF 10A and oral cancer cells with and without US treatment. Scale bar: 10 μm. (E, F) Bar graph showing the number and area of mature focal adhesions (stained by paxillin) in MCF 10A and oral cancer cells with and without US treatment. Two-way ANOVA followed by Tukey's multiple comparisons test, n > 100 cells from two different patient samples. (G) Time-lapse images of paxillin in MDA-MB-231 cells transfected with mApple-paxillin plasmid with and without US treatment. The white dotted line denotes the cell periphery. Scale bar: 10 μm. (H) The corresponding line graph showing mApple paxillin intensity at different time points with and without US treatment. Two-way ANOVA followed by Tukey's multiple comparisons test, n > 50 FAs from two independent experiments. In all experiments, ns: non-significant, *∗p* ​< ​0.05, *∗∗p* ​< ​0.01, *∗∗∗p* ​< ​0.001, and *∗∗∗∗p* ​< ​0.0001.

Recent studies have demonstrated that low-frequency US selectively kills different types of cancer cells due to their altered biomechanical properties [17,[19], [20], [21]]. To study the effect of low-frequency US on oral cancer growth, cells were treated with US treatment parameters (39 kHz frequency, 50% duty cycle, 50 kPa US pressure) up to 2 h, as previously reported [19]. Interestingly, we observed a significant reduction in spreading area (a two-fold reduction) and aspect ratio, as well as disassembly of F-actin organization in cancer cells upon US treatment (Fig. 2A, B, C). In stark contrast, normal cells did not display visible changes in spreading area, aspect ratio and F-actin organization upon US treatment.

Since US treatment caused a reduction in cancer cell size and aspect ratio, we focused on focal adhesions (FAs), which are present at the cell-extracellular matrix (ECM) interface. The FAs are integrin receptor-based adhesions that anchor cells to the ECM, promote cell spreading and convert biomechanical signals into biochemical signals via mechanotransduction [31,32]. It has been reported that mature FAs with an area >0.85 μm^2^ regulate cell spreading under tension [33,34]. To assess the effect of US treatment on FAs, we examined paxillin, an FA protein. A notable reduction in mature FA number and size was observed upon US treatment (Fig. 2D, E, F). Interestingly, MCF 10A normal cells showed an increase in mature FA numbers and sizes after US treatment. A plausible explanation for the reduction in FA number and size in cancer cells is the selective entry of calcium ions through Piezo1 mechanosensitive channels upon US treatment [35]. Calcium-activated calpain 2 has been shown to disassemble FAs by cleaving several FA proteins, including talin, which is essential for FA maturation [36]. To test this, highly metastatic breast cancer cells (MDA-MB-231) were transfected with mApple-paxillin and subjected to US treatment. Interestingly, a significant disassembly of peripheral FAs was observed in US-treated cancer cells compared with untreated breast cancer cells (Fig. 2G and H). Altogether, these results suggest that low-frequency US treatment selectively reduces cancer cell spreading by disassembling FAs without significantly impairing the spreading of normal cells.

### 3.3 Low-frequency ultrasound suppresses oral cancer cell migration and invasion by disrupting contractility

**Fig. 3:**
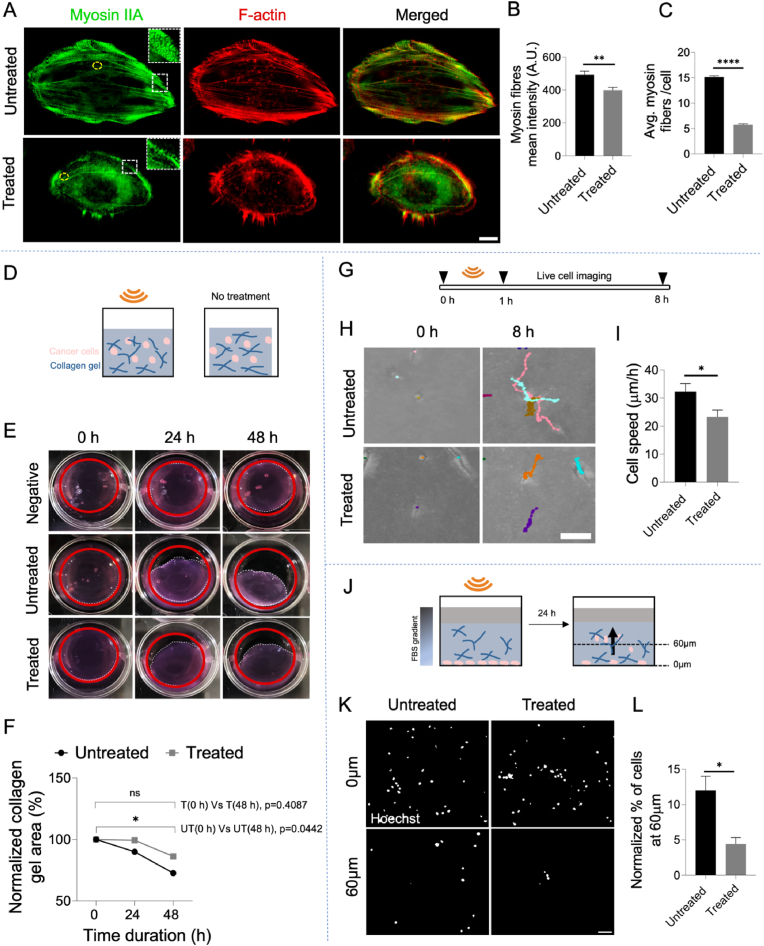
Low-frequency ultrasound suppresses oral cancer cell migration and invasion by disrupting contractility. (A) Representative images of myosin IIA and F-actin of primary oral cancer cells with and without US treatment. Scale bar: 10 μm. (B) Bar graph showing the mean intensity of myosin IIA fibres with and without US treatment. Two-sided, unpaired Student's t-test, n > 100 cells from two different patient samples. (C) Bar graph showing the average number of myosin fibers per cell with and without US treatment. Two-sided, unpaired Student's t-test, n > 100 cells, two different patient samples. (D) Schematic of collagen gel contraction assay. (E) Representative images of 3D collagen gel embedded with oral cancer cells at different time points with and without US treatment. The red line denotes the area of an individual well. The dotted white line indicates the area of the collagen gel. (F) Line graph showing changes in cancer cell-embedded collagen gel with and without US treatment at varying time points. Two-way ANOVA followed by Tukey's multiple comparisons test, n = 2 independent experiments using triplicate gels per condition and using cells from two patient samples (G) The timeline of the cancer cell migration assay. (H) Representative brightfield images of cell tracks at 0 h and 8 h with and without US treatment. Scale bar: 100 μm. (I) Bar graph showing the cell speed with and without US treatment. Two-sided, unpaired Student's t-test, n > 50 cells, from two different patient samples. (J) Schematic of 3D invasion assay. (K) Representative images of primary oral cancer cells stained with Hoechst (white) at 0 and 60 μm height of the collagen matrix with and without US treatment. Scale bar: 100 μm. (L) Bar graph showing the normalized percentage of cells at 60 μm height of the collagen matrix with and without US treatment after 24 h. Two-sided, unpaired Student's t-test, n > 100 cells from two different patient samples. In all experiments, ns: non-significant *∗p* ​< ​0.05, *∗∗p* ​< ​0.01, *∗∗∗p* ​< ​0.001, and *∗∗∗∗p* ​< ​0.0001. (For interpretation of the references to colour in this figure legend, the reader is referred to the Web version of this article.)

We recently reported that low-frequency ultrasound treatment significantly suppressed CAF contractility by disassembling stress fibers and inhibiting their colocalization with α-SMA [21]. Since CAFs and cancer cells exhibit similar biomechanical properties [37,38], we investigated the effect of US treatment on the contractility of oral cancer cells. First, we assessed the expression levels and spatial distribution of non-muscle myosin IIA in US-treated and untreated cancer cells. Surprisingly, a visible disassembly of myosin IIA fibers was observed upon US treatment (2 h) compared to the untreated samples (Fig. 3A and Fig. S5A). In particular, a significant reduction in myosin fiber intensity, the number of intact myosin fibers and cell population showing disassembled myosin fibers was observed in US-treated cells (Fig. 3B, C and Fig. S5B).

Cancer cells use actomyosin contractility to perform several activities, such as migration, invasion and ECM remodeling [39,40]. Since there is a notable disruption in the actomyosin cytoskeleton of cancer cells upon US treatment, we tested whether similar US treatment affects their contractility and migration ability. Firstly, the 3D collagen gel contraction assay was performed. The US-treated and untreated cancer cells were embedded in a collagen gel and analyzed for gel contraction over time (Fig. 3D), a process dependent on myosin contractility. ∼30% reduction in gel contraction was observed after 48 h in treated cells (Fig. 3E and F). In contrast, untreated cancer cells exhibited a notable gel contraction (58% normalized gel area) over a similar time period. Next, cancer cells were treated with ultrasound for 1 h, and their migration ability was assessed for the next 8 h (Fig. 3G). A drastic reduction in migration speed was noticed in treated cells (23 μm/h) compared to untreated cells (33 μm/h) (Fig. 3H and I). To further investigate the effect of US treatment on the 3D invasion ability of cancer cells, cells were embedded in 3D collagen matrices (Fig. 3J). A significant reduction in the number of cells invading to a height of up to 60 μm in collagen matrices was observed after 24 h in US-treated oral cancer cells (Fig. 3K and L). Altogether, these results demonstrate the effectiveness of US treatment in suppressing the migration and contraction ability of oral cancer cells, which contribute to cancer invasion.

### 3.4 Low-frequency ultrasound causes mechanoptosis in patient-derived oral cancer cells

**Fig. 4:**
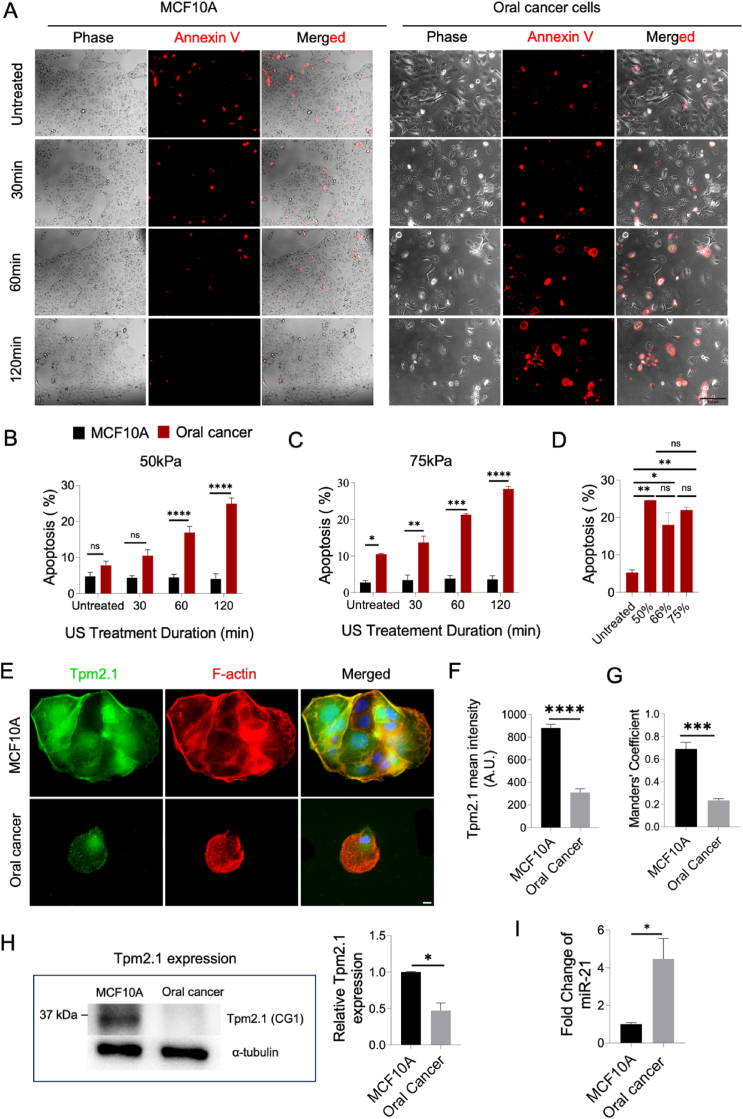
Ultrasound-mediated mechanical force promotes mechanoptosis in patient-derived oral cancer cells. (A) Representative images showing annexin V-positive apoptotic cells from normal breast epithelial cells (MCF 10A) and primary oral cancer cells at 39 kHz frequency and 50 kPa pressure with varying treatment duration. Scale bar: 150 μm. (B, C) Bar graph showing the level of apoptosis in MCF 10A and oral cancer cells at different pressure values, 50 kPa and 75 kPa, respectively, at different time durations. Two-way ANOVA followed by Tukey's multiple comparisons test, n > 5000 cells, data are representative of four independent experiments. (D) Bar graph showing the level of apoptosis in oral cancer cells at different duty cycles (50, 66, and 75%) at 50 kPa for 120 min. One-way ANOVA followed by Tukey's multiple comparisons test, n > 2000 cells, data are representative of three independent experiments. (E) Tpm2.1 and F-actin-stained images of MCF 10A and oral cancer cells. Scale bar: 10 μm. (F) Bar graph showing Tpm2.1 mean intensity of MCF 10A and oral cancer cells. Two-sided, unpaired Student's t-test, n > 100 cells from two different patient samples. (G) Bar graph showing F-actin and Tpm2.1 colocalization in MCF 10A and oral cancer cells. Two-sided, unpaired Student's t-test, n > 100 cells from two different patient samples. (H) Western blot results and corresponding bar graph exhibiting Tpm2.1 expression levels in MCF 10A and oral cancer cells. Two-sided, unpaired Student's t-test, N = 2 independent experiments using two different patient samples. (I) qRT-PCR results showing miR-21 expression level in MCF 10A and oral cancer cells. Two-sided, unpaired Student's t-test, N = 2 independent experiments from two different patient samples. In all experiments, ns: non-significant, *∗p* ​< ​0.05, *∗∗p* ​< ​0.01, *∗∗∗p* ​< ​0.001, and *∗∗∗∗p* ​< ​0.0001.

Recent reports have demonstrated that low-frequency US treatment causes selective apoptosis (mechanoptosis) of cancer cells *in vitro* and *in vivo*, without harming normal cells [17,[19], [20], [21]]. Molecular mechanistic studies have revealed that many tumor cell types exhibit aberrant mechanosensing due to a lack of mechanosensory cytoskeletal proteins, such as Tpm2.1, and undergo mechanoptosis [21,22]. To test whether US treatment induces mechanoptosis in oral cancer cells, cells were subjected to US treatment with different US pressures (50 kPa and 75 kPa), and the apoptosis level was measured using annexin V staining (Fig. 4A and Fig. S6A). Notably, higher levels of apoptosis (∼25% and 28%) were observed after 2 h of US treatment at 50 and 75 kPa, respectively (Fig. 4B and C). In stark contrast, a negligible level of apoptosis (∼5%) was observed in normal MCF10A and patient-derived oral epithelial cells (Fig. S6B), indicating a differential response to US treatment. Next, to assess the effect of the US duty cycle, cancer cells were treated at different duty cycles (50%, 66% and 75%). No prominent change in apoptosis levels was observed despite varying the US duty cycle (Fig. 4D). To rule out that apoptosis is not caused by ultrasound-induced thermal cavitation, the temperature of the cell culture media was monitored during US treatment. The temperature was maintained at 25^0^C by keeping the setup on thermal plates as shown in (Fig. S2A). A minimal change of ±1.5 °C was observed during the 2 h of treatment, indicating that thermal cavitation did not contribute to mechanoptosis (Fig. S2B).

**Fig. 5:**
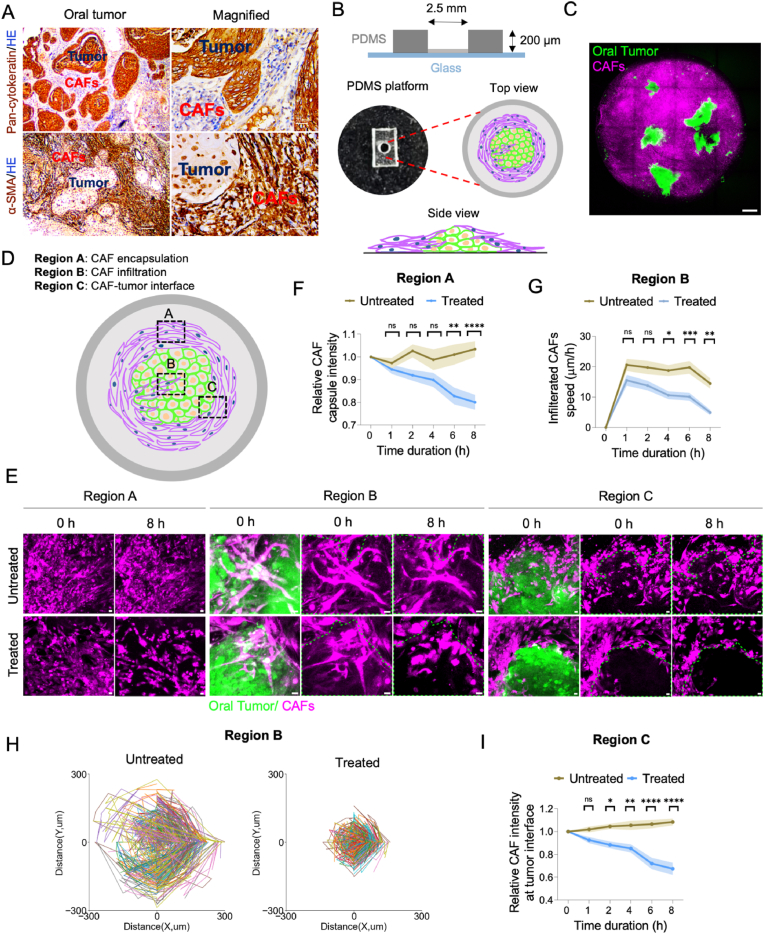
Low-frequency ultrasound disrupts the 3D organization of the oral tumor. (A) Representative H & E images showing α-SMA (CAF marker) and pan-cytokeratin (oral cancer cell) staining. Scale bar: 10x (left) and 20x (right). (B) Schematic of the PDMS well platform mimicking oral tumor organization. (C) Representative image of coculture of CAFs (magenta) and oral cancer cell aggregates (green) on PDMS well platform. Scale bar: 500 μm. (D) Schematic illustration of the regions of interest (ROI) in the platform: Region A: CAF encapsulation, Region B: CAF infiltration, Region C: CAF-tumor interface. (E) Representative images of cancer cells and CAFs in different regions at 0 h and 8 h, with and without US treatment (1 h). Scale bar: 20 μm. (F) Line graph showing the relative CAF intensity at region A at different time points with and without US treatment. n > 50 ROI from three different patient samples. (G) Line graph showing the speed of infiltrated CAFs in the tumor core (region B) at different time points with and without US treatment. n > 100 cells from three different patient samples. (H) Tracks of infiltrated CAFs in the tumor core (region B) for a duration of 8 h with and without US treatment. n > 50 ROI from three different patient samples. (I) Line graph showing the relative CAF intensity at region C at different time points with and without US treatment. n > 50 ROI from three different patient samples. In all experiments, a Two-way ANOVA followed by Tukey's multiple comparisons test, ns: non-significant, *∗p* ​< ​0.05, *∗∗p* ​< ​0.01, *∗∗∗p* ​< ​0.001, and *∗∗∗∗p* ​< ​0.0001. (For interpretation of the references to colour in this figure legend, the reader is referred to the Web version of this article.)

Recent reports have shown that Tpm2.1-depleted cancer cells and CAFs, or Tpm2.1 depletion in normal cells, make them vulnerable to US treatment and cause mechanoptosis [21,22]. Therefore, we tested the Tpm2.1 expression levels in oral cancer cells. In line with previous studies, lower levels of Tpm2.1 were observed in immunofluorescent images of oral cancer cells compared to normal cells (Fig. 4E and F). A significant reduction in the colocalization of F-actin and Tpm2.1 was observed in cancer cells compared to the normal cells (Fig. 4G). Additionally, western blot analysis confirmed a significant reduction in Tpm2.1 level in cancer cells compared to normal cells (Fig. 4H), highlighting that Tpm2.1-depleted oral cancer cells become mechanically vulnerable and undergo mechanoptosis.

To further confirm the role of Tpm2.1 on mechanoptosis, Tpm2.1 siRNA was used to knock down (KD) the Tpm2.1 variant in wild-type MCF 10A cells Fig. S7A). Western blot analysis showed a significant reduction in Tpm2.1 levels in KD cells compared with wild-type cells (Fig. S7B). Moreover, wild-type MCF 10A cells were cultured in oral cancer cell-conditioned media (OCM) for two days to test the effect of oral cancer cell secretome on Tpm2.1 expression levels in MCF 10A cells. Intriguingly, a notable reduction in Tpm2.1 expression levels was observed in MCF 10A grown in OCM and in oral cancer cells compared with wild-type MCF 10A cells (Fig. S7C). Next, we assessed the mechanoptosis level in these cell types. A significant increase in mechanoptosis was observed in Tpm2.1 KD MCF 10A cells and MCF 10A cells grown in OCM compared to wild-type MCF 10A cells (Fig. S7D), suggesting a crucial role of Tpm2.1 in optimal mechanosensing of external mechanical forces and in suppressing mechanoptosis in normal cells.

It is well known that oncogenes downregulate tropomyosin expression in cells during cancer development by activating miR-21 [41]. In particular, increased miR-21 expression has been observed in patients with oral squamous cell carcinoma, which is associated with a poor prognosis [42,43]. Similarly, our qRT-PCR analysis revealed a four-fold increase in miR-21 expression levels in oral cancer cells compared to normal cells (Fig. 4I), suggesting that miR-21 secreted by oral cancer cells downregulates Tpm2.1 levels and initiates mechanoptosis upon US treatment.

### 3.5 Ultrasound treatment disrupts *in vivo* mimicking oral cancer cell-CAF 3D organization

CAFs located in the tumor microenvironment (TME) secrete excessive ECM and form a dense capsule-like structure around the tumor core [44], inhibiting immune cells and chemotherapeutic drugs from infiltrating the tumor core [[45], [46], [47]]. Additionally, CAF infiltration into the tumor core has been shown to lead to tumor compartmentalization [48] and increase tumor aggressiveness [49]. Thus, to study the oral tumor architecture, immunohistochemistry was performed on patient-derived oral tumor samples. Several tumor islands (stained by pan-cytokeratin) were found surrounded by a thick capsule-like structure formed by elongated CAFs (stained by α-SMA) (Fig. 5A). A PDMS elastomer platform was developed to mimic the *in vivo* organization of oral tumors and to assess the effect of US treatment on the interaction between cancer cells and CAFs (Fig. 5B). Patient-derived oral tumor aggregates (300-500 μm size) and CAFs were labelled with green and red fluorescent live cell trackers, respectively and cocultured as described in the materials section to mimic oral TME (Fig. 5C and Fig. S8). Three different regions of the coculture were considered to study the effect of US treatment on CAF-CAF interactions within CAF's encapsulation (Region A), infiltrated CAFs into the tumor core (Region B) and tumor cell-CAFs interactions at the tumor-CAF interface (Region C) (Fig. 5D). The cocultured cells were subjected 1 h of US treatment, followed by 8 h of live-cell imaging (Fig. 5E). Surprisingly, an 80% reduction in CAFs capsule intensity (Region A) was observed after 8 h upon US treatment, whereas no drastic reduction in CAFs intensity was noticed in untreated samples (Fig. 5E and F). Next, we examined the speed of infiltrated CAFs in the tumor core. In untreated samples, CAFs invaded the tumor core at approximately 20 μm/h for 6 h, after which the speed decreased to 15 μm/h at 8 h (Fig. 5G). In stark contrast, the speed of CAF invasion substantially reduced from 15 μm/h (1 h) to 5 μm/h (8 h) in US-treated cells. Similarly, the distance covered by infiltrated CAFs in the tumor core upon US treatment was halved compared to the distance covered by infiltrated CAFs without treatment (Fig. 5E–H). Furthermore, we checked the effect of the US treatment on tumor cell-CAFs interactions at the tumor-CAF interface (Region C). A remarkable reduction in the CAF area at the interface was observed upon US treatment, exposing the tumor core, as indicated by a decrease in CAF intensity (∼33% reduction) (Fig. 5E–I). On the other hand, a minor increase in CAF area and intensity was noticed at the interface in untreated cells. Surprisingly, these untreated CAFs at the interface infiltrated the tumor core, indicating an early sign of tumor compartmentalization. (Fig. 5E). Overall, these results demonstrated that the US treatment not only disrupts tumor-CAFs interactions but also CAF-CAF interactions, leading to loosening of the stromal barrier around the tumor core that could enhance drug permeability.

## 4 Discussion

This study presents a mechanistic investigation of the effect of ultrasound-generated mechanical forces on patient-derived oral cancer cell apoptosis at the cellular level, with a broader focus on the integrity of oral tumor tissue organization. In particular, optimized US treatment causes selective mechanoptosis of patient-derived oral cancer cells without damaging normal epithelial cells in *ex vivo* studies. The results further reveal that US treatment caused a similar level of apoptosis despite the patient heterogeneity with varying cancer stages and tumor origin. At the subcellular level, US treatment causes a visible reduction in FA size and numbers, leading to shrunken morphology in cancer cells. We observe that US treatment significantly disrupts the contractility of oral cancer cells by disassembling myosin IIA fibers, which cancer cells use to support cancer progression. Our results indicate that the oral cancer cells are mechanically vulnerable to physiologically relevant US treatment due to the absence of the mechanosensory cytoskeletal protein Tpm2.1. We further show that US-mediated mechanical forces disrupt the integrity of cancer cell-CAFs in coculture, leading to loosening of the CAF's capsule-like structure around the tumor core.

FA structures facilitate mechanotransduction at the cell-ECM interface, i.e., conversion of external biophysical signals into biochemical signals, and transmit these signals to the nucleus [50,51]. Our findings showed that US treatment disrupts mature FAs in oral cancer cells and reduces their cell area, whereas normal epithelial cells show increased formation of mature FAs. A plausible explanation for the reduction in FA size and area in cancer cells is the entry of calcium ions through Piezo1 mechanosensitive channels present on the cell membrane upon US treatment [35]. Calcium-activated calpain has been shown to disassemble FAs by cleaving several FA proteins, including talin, which is essential for FA maturation [36]. Taken together, the results indicate that normal cells withstand physiologically relevant US treatment and form mature FAs, whereas oral cancer cells showed reduced FAs, leading to shrunken morphology.

Cancer cells mainly rely on actomyosin contractility for 3D invasion and metastasis [52]. Surprisingly, our findings revealed that US treatment significantly disrupts the contractility of oral cancer cells by disassembling myosin IIA fibers. 3D migration assays and collagen gel contraction assay, which are reliable indicators of actomyosin contractility, showed a significant reduction in migration and contraction ability of oral cancer cells. Further, the 3D gel invasion assay showed that US treatment significantly reduces the cancer cell invasiveness. Collectively, these results suggest that US treatment suppresses oral cancer cell migration and invasion by targeting myosin contractility.

Tpm2.1 is a rigidity-sensing protein that controls cellular contractility by regulating actin filament-myosin IIA interactions [22,53]. Our findings reveal that the mechanical vulnerability of oral cancer to US treatment depends on Tpm2.1 and induced mechanoptosis. In addition, depleting Tpm2.1 in normal epithelial cells increased vulnerability to US treatment (Fig. S7). Surprisingly, ‘The Cancer Genome Atlas’ **(**TCGA) database demonstrated a notable reduction in TPM2 gene (which encodes Tpm2.1) expression levels in 19 different cancer types, including head and neck cancer, compared to their matched normal tissue counterparts [54]. It is important to note that oral cancer is one of the important subtypes of head and neck cancer. Previous reports have shown that several cancer cell types, including oral cancer cells, secrete miR-21, which downregulates key tumor suppressor genes, including TPM2 [43,[55], [56], [57], [58]]. Similarly, we observed a notable reduction in Tpm2.1 expression in normal cells grown in oral cancer cell-conditioned media (Fig. S7C), strongly suggesting that miR-21 suppresses Tpm2.1 and promotes mechanoptosis in oral cancer cells.

**Fig. 6:**
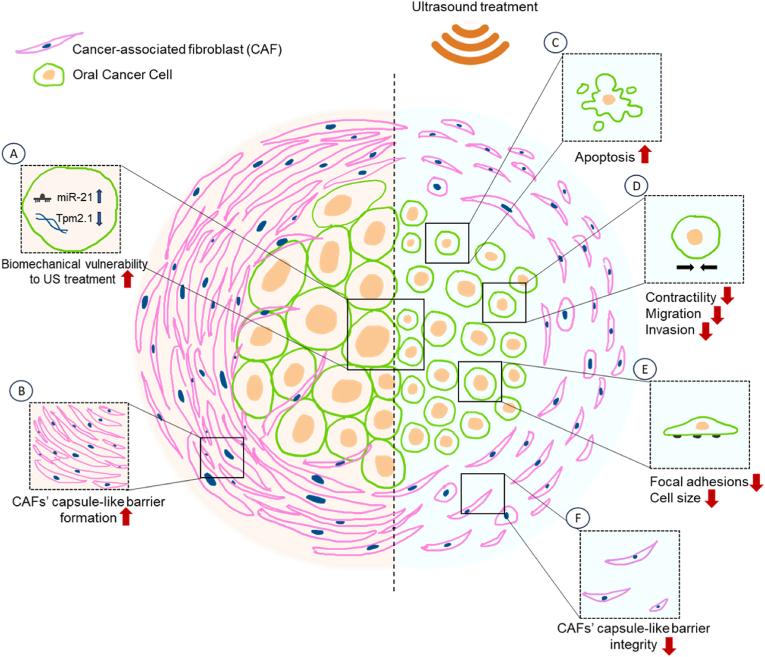
Proposed model of US-mediated disruption of oral cancer cell-CAF 3D organization. (A) Oral cancer cells exhibit higher miR-21 expression, which downregulates Tpm2.1, thereby increasing their vulnerability to US treatment. (B) During cancer progression, CAFs form a capsule-like barrier around the tumor core, preventing immune cell infiltration and drug penetration. Due to biomechanical vulnerability, US treatment promotes (C) oral cancer cell mechanoptosis, (D) suppression of myosin-mediated contractility, migration, and invasion, (E) induces shrunken morphology through focal adhesion disassembly, and (F) compromises the integrity of the CAF's capsule-like barrier.

The oral tumor core is surrounded by a dense capsule of CAFs and ECM secreted by these cells [44,59]. This capsule-like barrier not only prevents immune cells and drugs from entering the tumor but also generates mechanical stress within the tumor, leading to a more aggressive tumor [49,60,61]. In recent years, targeting CAF's capsule-like barrier has emerged as an attractive approach to enhance therapeutic efficacy [62]. To model this architecture, a PDMS-based coculture platform was developed and used to evaluate the effect of US treatment. US treatment markedly disrupted CAF-CAF and CAF-cancer cell interactions, reducing tumor core encapsulation, CAF infiltration speed and CAF accumulation at the tumor interface (Fig. 6). These results are consistent with our recent work, in which CAFs showed reduced contractility upon US treatment [21]. These US treatment effects collectively loosened the CAF-mediated barrier, exposing the tumor core. Although our PDMS platform is limited by its simplicity, incorporating only cancer cells and CAFs and excluding other cell types, it nevertheless offers a unique approach to studying CAF-CAF interactions within a capsule-like structure, infiltrated CAFs in the tumor core, and tumor cell-CAFs interactions at the tumor interface upon US treatment.

Our simple 3D coculture elastomer model occupies an intermediate position between 2D platforms and more complex 3D platforms, such as organoid systems, organ-on-a-chip platforms and cell-embedded 3D bioprinted platforms. Unlike organoids, which recapitulate tissue architecture, functionality and heterogeneity, our reductionist coculture platform focuses on a defined tumor core-stromal cell microenvironment to quantify tumor cell survival and the CAF's ability to encapsulate and infiltrate the tumor core under ultrasound treatment. Moreover, such a simpler model enables high-resolution real-time imaging to assess the effects of ultrasound-mediated mechanical stimulation on interactions between tumor cells and tumor-associated cells in a controlled and spatiotemporal manner. While we acknowledge the limitations of this model due to the absence of other tumor-associated cells (immune cells and endothelial cells), it complements rather than replaces validation assays based on organoid and animal studies. Another limitation of this study is the inadequate availability of matched healthy oral mucosal epithelial cells as controls for the direct comparison. Thus, future studies should include adjacent normal oral mucosa or healthy tissues obtained during clinical procedures, such as crown-lengthening or third molar extraction, to allow accurate characterization and analysis of cancer-specific biomechanical responses upon mechanical perturbation.

Altogether, our study demonstrated a unique approach of using non-invasive low-frequency US treatment to target oral cancer cells. However, an in-depth understanding of the mechanistic effects of US treatment on the oral TME using complex 3D platforms and *in vivo* models will be required before it can be used to develop personalized treatment for patients who typically present with fewer comorbidities. Given the anatomical accessibility, US treatment can be extended to other superficial cancers like breast cancer and skin cancer.

## 5 Conclusion

We demonstrated that optimized ultrasound-mediated mechanical forces selectively promoted mechanoptosis in oral cancer cells from different oral cavity sites of multiple patients at varying cancer stages. The mechanical vulnerability of oral cancer cells was attributed to increased miR-21 expression, which reduced Tpm2.1, a cytoskeletal mechanosensory protein responsible for optimal mechanosensing. Furthermore, the effect of the US treatment on the cancer cell contractility, migration and invasion was due to the disassembly of myosin IIA fibers. Studies using the 3D elastomeric platform, which mimics *in vivo* oral TME, demonstrated that US treatment disrupts the CAFs' capsule-like structure around the tumor core, thereby compromising the barrier that protects the tumor core. In addition, US treatment compromises CAFs’ invasion into the tumor core, preventing tumor compartmentalization. With greater physiological relevance, our findings in patient-derived oral cancer cells could facilitate the clinical translation of low-frequency ultrasound treatment for oral cancer, overcoming current therapeutic limitations.

## CRediT authorship contribution statement

**Rashmita Luha:** Conceptualization, Data curation, Investigation, Methodology, Software, Validation, Visualization, Writing – original draft, Writing – review & editing. **Gomathi Sankar:** Data curation, Methodology, Writing – review & editing. **Akshay Kumar:** Investigation, Methodology, Writing – review & editing. **Alka Kumari:** Methodology. **Ketan Kulkarni:** Methodology. **Rudra Pratap:** Supervision. **Aravind Kapali:** Resources. **Ajay Tijore:** Conceptualization, Supervision, Writing – original draft, Writing – review & editing.

## Declaration of competing interest

The authors declare that they have no known competing financial interests or personal relationships that could have appeared to influence the work reported in this paper.

## Data Availability

Data will be made available on request.
