# Supplementary material for: Revealing biomechanical vulnerabilities in oral cancer cells using 3D coculture platform and low-frequency ultrasound
Source: Mater Today Bio. 2026 Jun 19;39:103375. doi: 10.1016/j.mtbio.2026.103375 (PMC13315667; doi:10.1016/j.mtbio.2026.103375)
Supplement: Multimedia component 1 [file mmc1.docx]

Supporting Information

**Revealing biomechanical vulnerabilities in oral cancer cells using 3D coculture platform and low‑frequency ultrasound**

Rashmita Luha^1^, Gomathi Sankar^1^, Akshay Kumar^2^, Alka Kumari^1^, Ketan Kulkarni^1^, Rudra Pratap^2,3^, Aravind Kapali^4^, Ajay Tijore^1^*

^1^Department of Bioengineering, Indian Institute of Science, Bangalore, India, 560012

^2^Centre for Nano Science and Engineering, Indian Institute of Science, Bangalore, India, 560012

^3^Plaksha University, Mohali, India, 140306

^4^Department of Surgical Oncology, M. S. Ramaiah Medical College & Hospitals, Bangalore, India, 560054

Correspondence: ajaytijore@iisc.ac.in


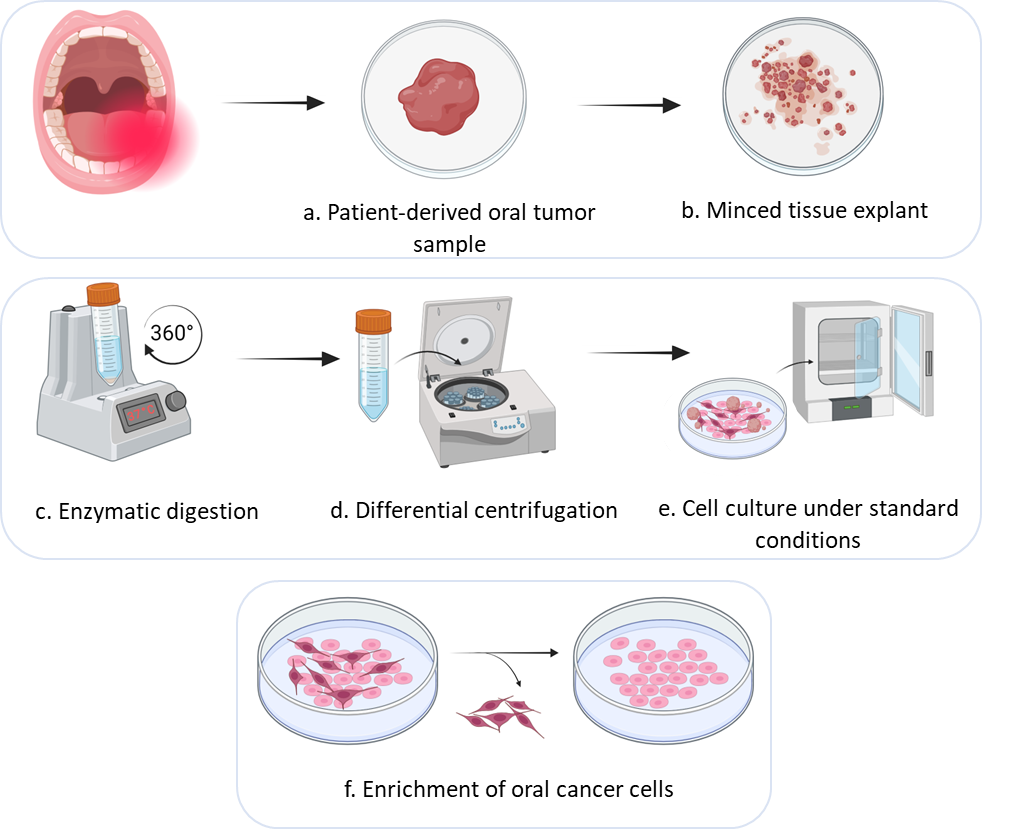


**Figure S1. Isolation of oral cancer cells from a patient-derived tumor sample.** Schematic illustrating the steps involved in isolating primary oral cancer cells from patient tumor samples.


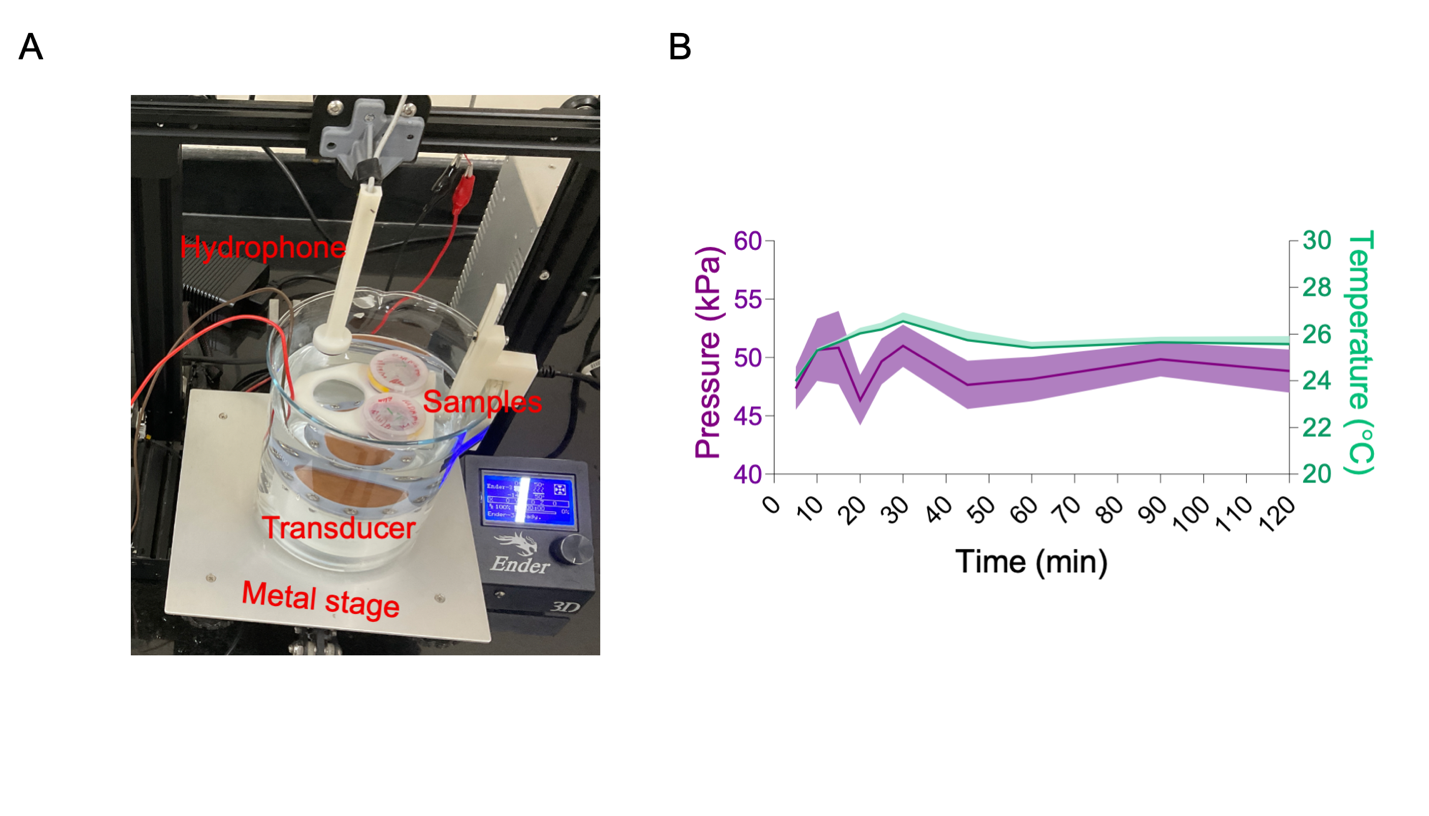
**Figure S2. Schematic showing a custom-made ultrasound device. (A)** Custom-built ultrasound device setup to apply optimized US-mediated mechanical forces on cells. **(B)** The dual-axis chart displays the pressure and temperature values for 120 min of US treatment (50% duty cycle, 39 kHz frequency, 50 kPa pressure), measured using a hydrophone and a thermostat, respectively, at the glass dish surface. Data are obtained using six independent experiments. Data show mean ± SEM.


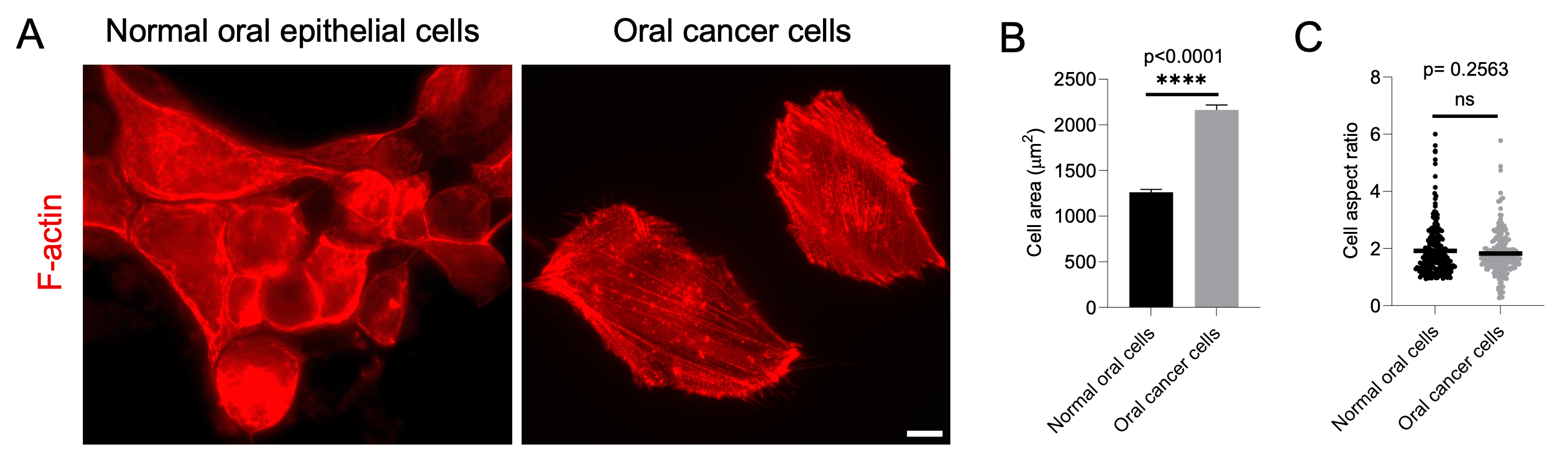


**Figure S3.** Morphological features of patient-derived healthy oral epithelial cells and oral cancer cells. **(A)** Representative F-actin-stained images of patient-derived normal oral epithelial cells and oral cancer cells. Scale bar: 10 μm. **(B, C)** Bar graph showing the cell area and aspect ratio of these cells, respectively. Two-sided, unpaired Student’s t-test, n > 100 cells from two different patient samples. Data show mean ± SEM. In all experiments, ns: non-significant, **p* ​< ​0.05, ***p* ​< ​0.01, ****p* ​< ​0.001, and *****p* ​< ​0.0001.


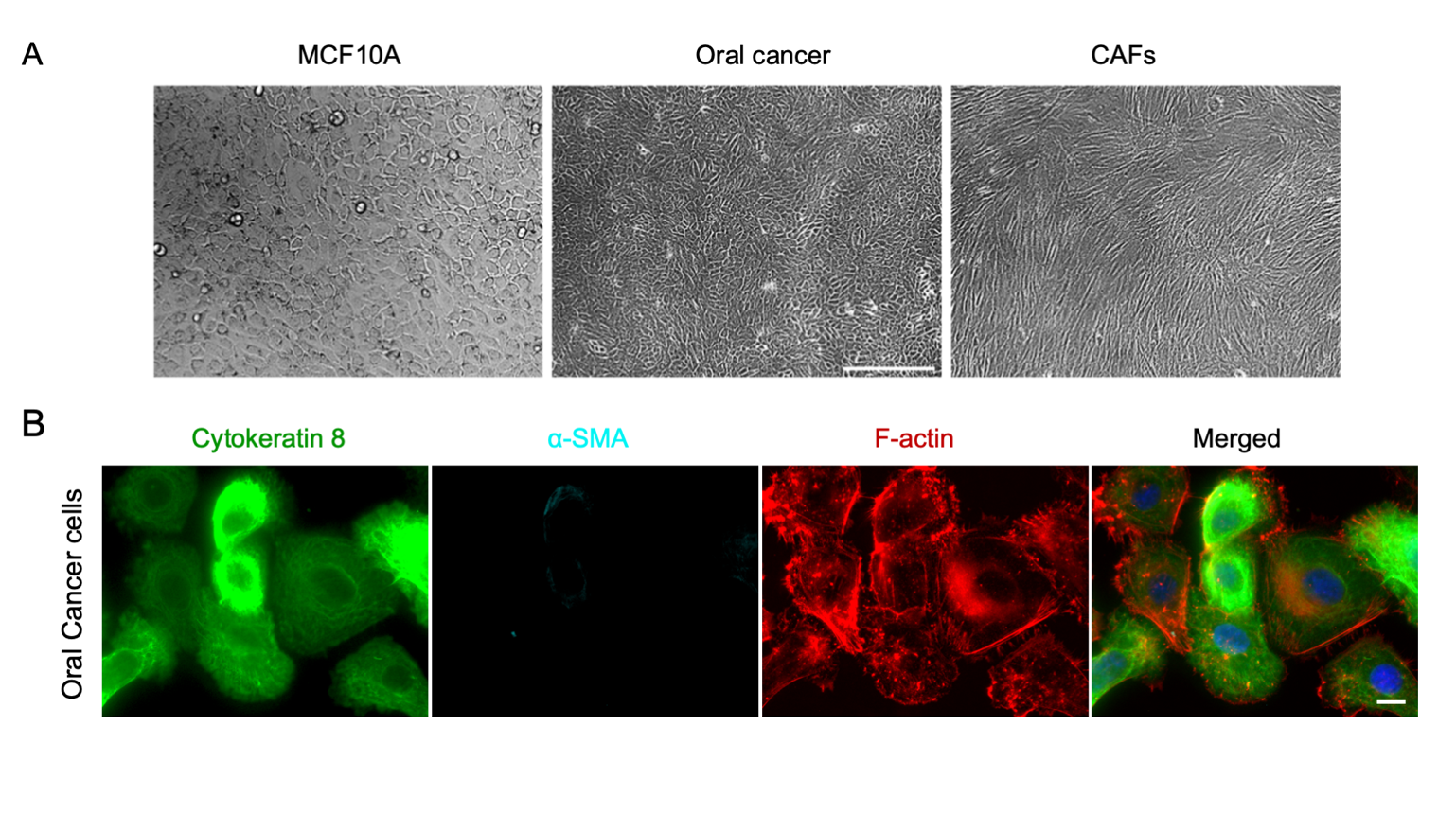
**Figure S4. Characterization of patient-derived oral cancer cells (A)** Representative brightfield images of normal breast epithelial cells (MCF-10A), primary oral cancer cells and CAFs. Scale bar: 300 µm **(B)** Representative immunofluorescence images of primary oral cancer cells stained for cancer cell marker (cytokeratin 8), CAF marker (α-SMA), and stress fiber (F-actin). Scale bar: 10 µm.

**
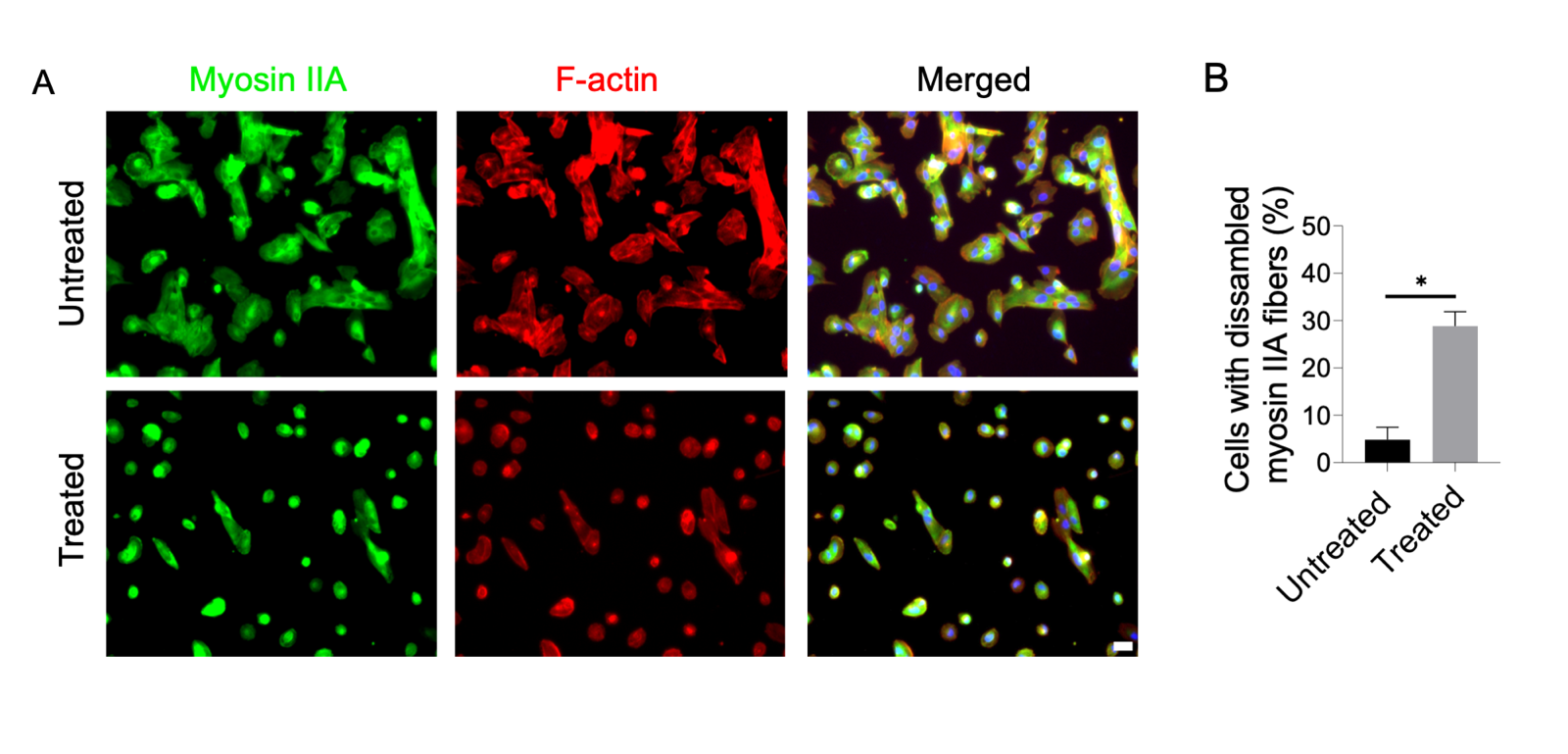
Figure S5. Ultrasound treatment disassembles the myosin IIA fibers in oral cancer cells.** (**A**) Representative immunofluorescence images of myosin IIA fibers with and without US treatment (50 kPa for 2 h). Scale bar: 100 μm. (**B**) Quantitative analysis of cancer cell population showing intact myosin IIA fibers with and without US treatment. Unpaired Student’s t-test, n>200 cells from two independent experiments, **p* < 0.05.


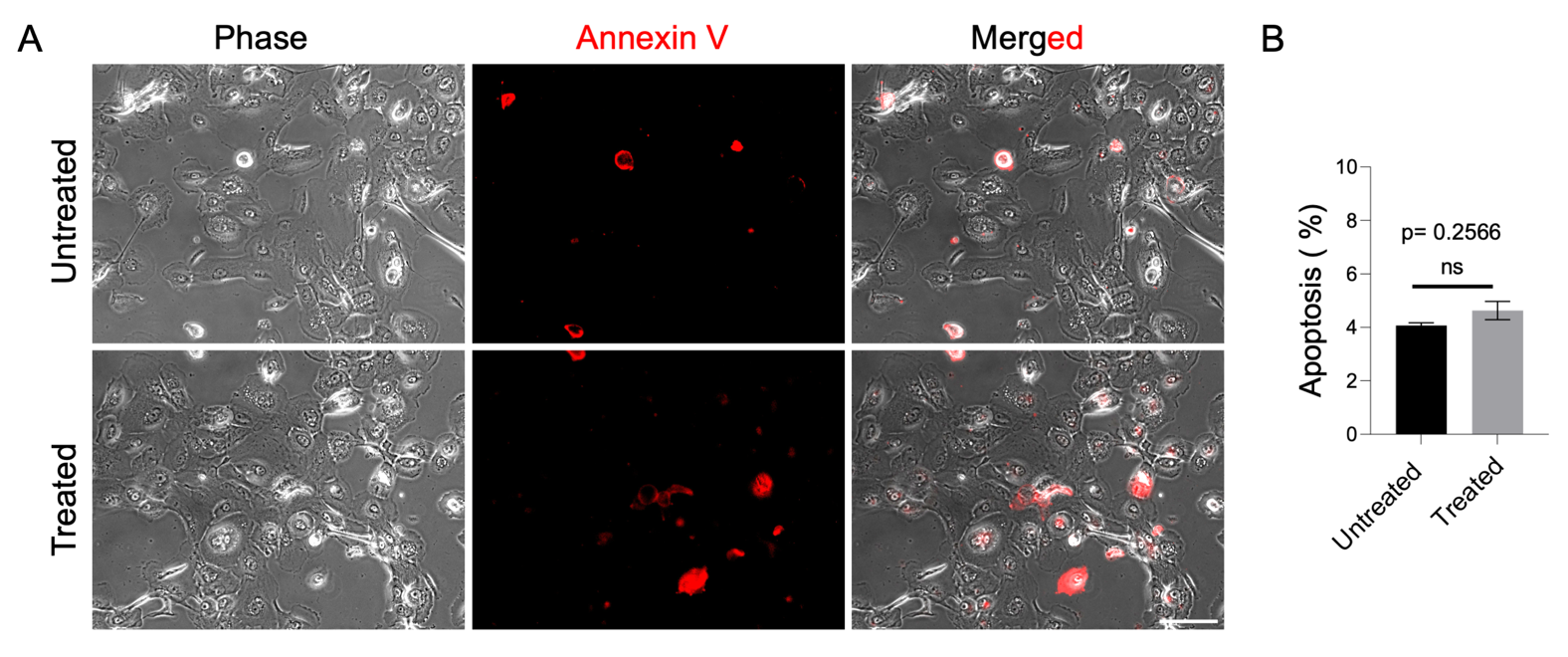


**Figure S6. Patient-derived healthy oral epithelial cells showed negligible apoptosis upon low-frequency ultrasound treatment (50 kPa)**. **(A)** Representative images showing annexin V-positive apoptotic cells in healthy oral epithelial cells with and without ultrasound treatment. Scale bar: 100 μm. **(B)** Corresponding bar diagram showing the apoptosis level in treated and untreated healthy cells. Two-sided, unpaired Student’s t-test, n > 400 cells, three individual experiments using two different patient samples. Data show mean ± SEM. In experiments, ns: non-significant and **p* ​< ​0.05.

**
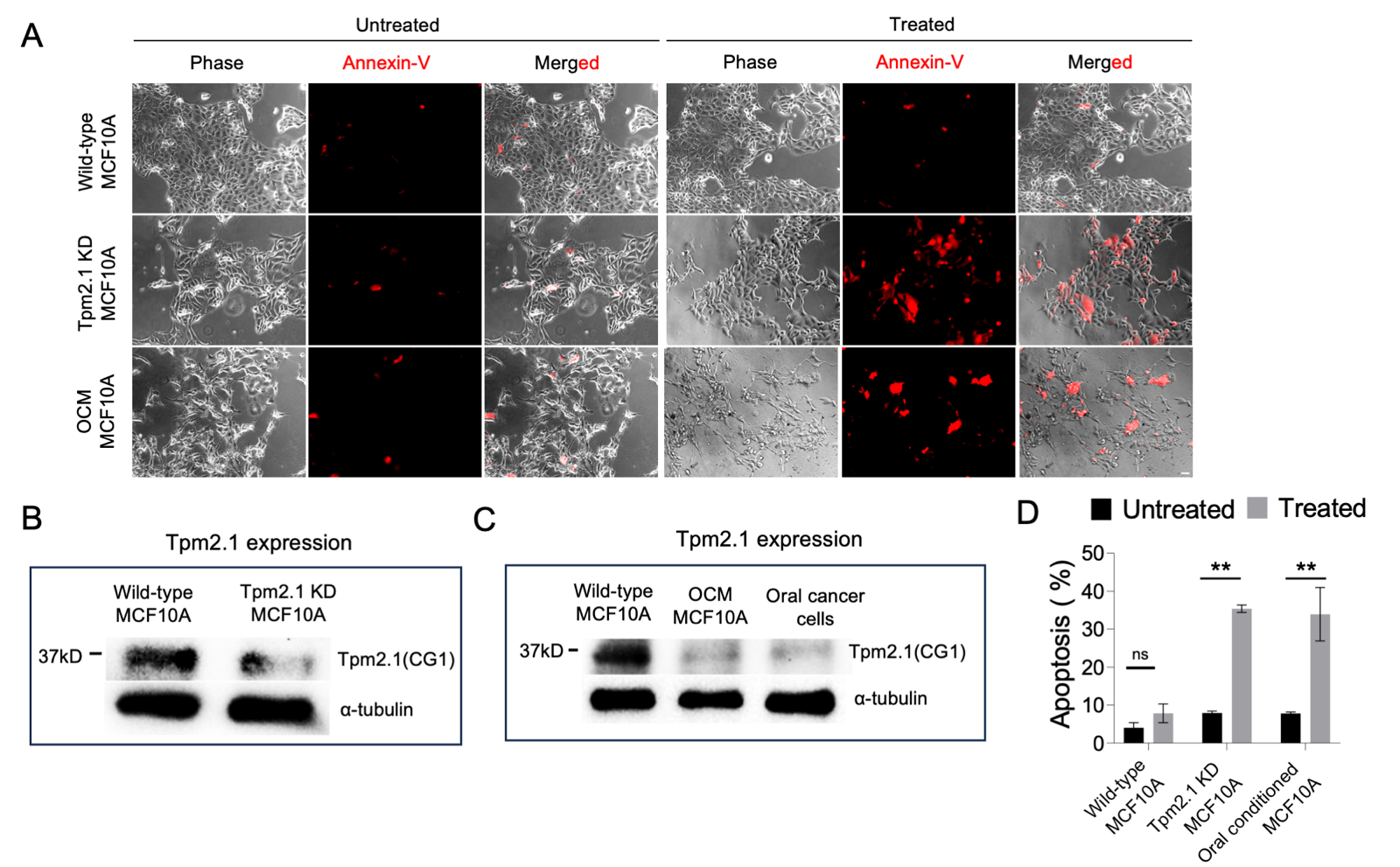
**

**Figure S7. Tpm2.1 knockdown (KD) in normal cells causes mechanoptosis. (A)** Representative images of Annexin V-stained wild-type MCF-10A, Tpm2.1 KD MCF-10A, and MCF-10A cells grown in oral cancer cell conditioned media (OCM MCF-10A) with and without US treatment (2h). Scale bar: 100 μm. **(B)** Western blot results showing Tpm2.1 expression level in wild-type and Tpm2.1 KD MCF-10A cells. **(C)** Western blot results showing Tpm2.1 expression levels in wild-type MCF-10A cells, OCM MCF-10A cells, and oral cancer cells. **(D)** Bar diagram illustrating the apoptosis level in various cell types with and without US treatment. Two-way ANOVA followed by Tukey’s multiple comparisons test, n>2000 cells from two independent experiments. ns: non-significant, ***p* < 0.01.


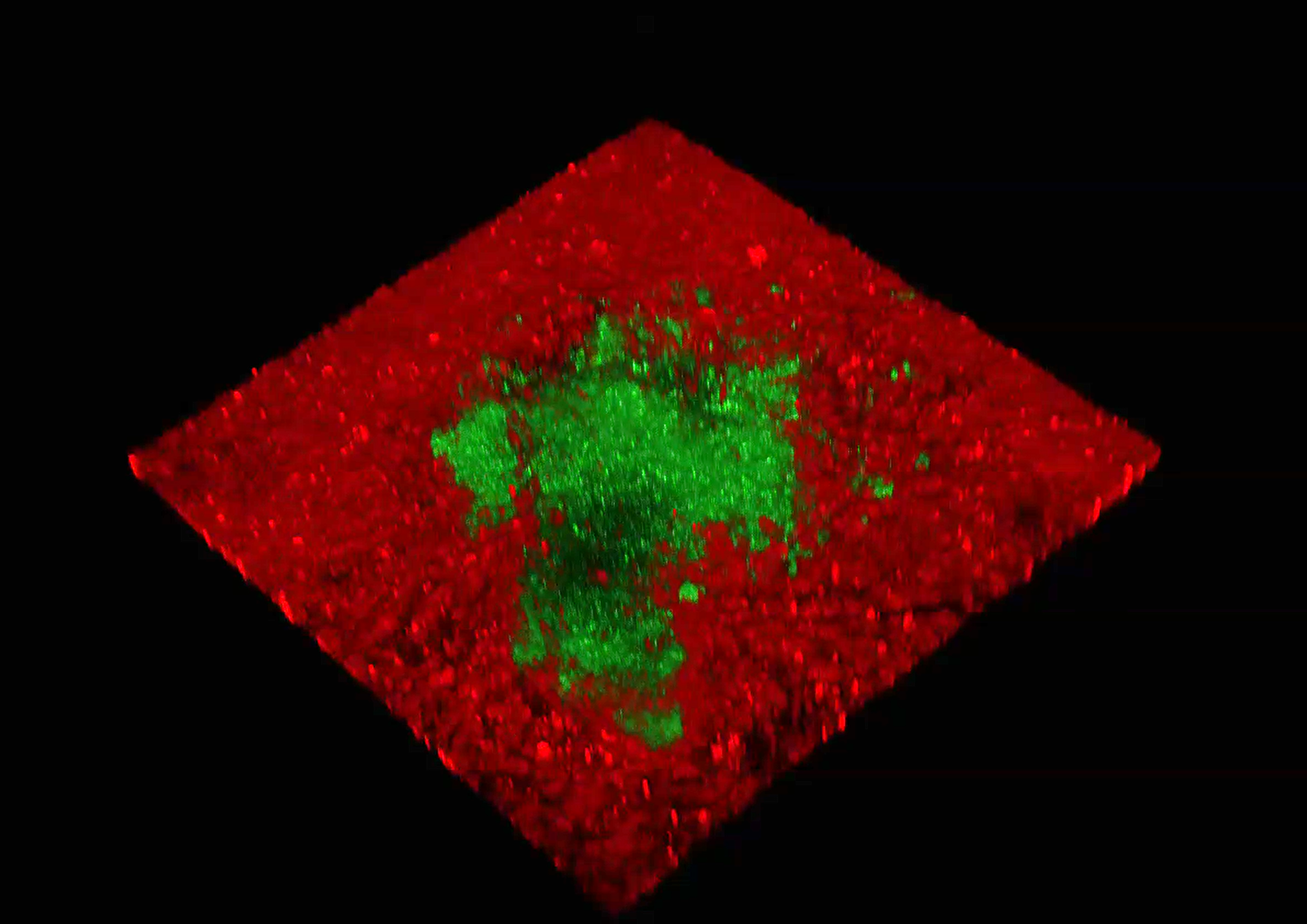


z

y

x

Tumor core

Cancer-associated fibroblasts (CAF)

**Figure S8.** 3D Representative image of a coculture of patient-derived oral cancer cell aggregates (green) and CAFs (red) on a PDMS well platform*.*

**Table S1.** Tumor sample details used in this study

| Patient no. | Gender | Age | Cancer Stage | Tumor region | Tobacco history | Figures |
| --- | --- | --- | --- | --- | --- | --- |
| 1 | M | 44 | cT2N1M0 | Buccal mucosa | 30 years | 1A, 1D, 4A |
| 2 | M | 66 | cT4N1M0 | Buccal mucosa | 50 years | 1A, 1D, 4A |
| 3 | M | 47 | cT2N1M0 | Buccal mucosa | 12 years | 2A, 2D, 3H |
| 4 | F | 68 | cT4N1M0 | Buccal mucosa | 50 years | 2A, 2D, 3H |
| 5 | F | 51 | cT4N1M0 | Alveolus | 15 years | 3A, 3K, 4E |
| 6 | F | 72 | cT4aN1M0 | Buccal mucosa | 40 years | 3A, 3K |
| 7 | F | 49 | cT2N0M0 | Buccal mucosa | 15 years | 3E, 4A |
| 8 | M | 60 | cT4N1M0 | Buccal mucosa | 30 years | 3E, 4A |
| 9 | F | 60 | cT3N0M0 | Tongue | 30 years | 4A |
| 10 | M | 39 | cT4aN2bM0 | Buccal mucosa | 15 years | 5A,5E |
| 11 | M | 36 | cT2N1M0 | Buccal mucosa | 20 years | 4E,5E |
| 12 | M | 42 | cT2N1M0 | Buccal mucosa | 25 years | 5A,5E |

**Table S2**. Primer sequences of miR-21 and U6 snRNA

| **Primer** | **Sequence** | |
| --- | --- | --- |
| U6 snRNA | Forward | 5′-GCGCGTAGCTTATCAGACTGA-3′ |
|  | Reverse | 5′-AGTGCAGGGTCCGAGGTATT-3′ |
| miR-21 | Forward | 5′-CTCGCTTCGGCAGCACA-3′ |
|  | Reverse | 5′-AACGCTTCACGAATTTGCGT-3′ |
